# Improved Mechanical Strength of Dicatechol Crosslinked MXene Films for Electromagnetic Interference Shielding Performance

**DOI:** 10.3390/nano13050787

**Published:** 2023-02-21

**Authors:** Soyeon Kim, Canh Minh Vu, Suehyeun Kim, Insik In, Jihoon Lee

**Affiliations:** 1 Department of IT Energy Convergence (BK21 FOUR), Korea National University of Transportation, Chungju 27469, Republic of Korea; 2 Advanced Institue of Science and Technology, The University of Da Nang, Da Nang 550000, Vietnam; 3 Department of Polymer Science and Engineering, Korea National University of Transportation, Chungju 27469, Republic of Korea

**Keywords:** MXene, dicatechol, crosslinking, mechanical properties, EMI shielding

## Abstract

Pristine MXene films express outstanding excellent electromagnetic interference (EMI) shielding properties. Nevertheless, the poor mechanical properties (weak and brittle nature) and easy oxidation of MXene films hinder their practical applications. This study demonstrates a facile strategy for simultaneously improving the mechanical flexibility and the EMI shielding of MXene films. In this study, dicatechol-6 (DC), a mussel-inspired molecule, was successfully synthesized in which DC as mortars was crosslinked with MXene nanosheets (MX) as bricks to create the brick-mortar structure of the MX@DC film. The resulting MX@DC-2 film has a toughness of 40.02 kJ·m^−3^ and Young’s modulus of 6.2 GPa, which are improvements of 513% and 849%, respectively, compared to those of the bare MXene films. The coating of electrically insulating DC significantly reduced the in-plane electrical conductivity from 6491 S·cm^−1^ for the bare MXene film to 2820 S·cm^−1^ for the MX@DC-5 film. However, the EMI shielding effectiveness (SE) of the MX@DC-5 film reached 66.2 dB, which is noticeably greater than that of the bare MX film (61.5 dB). The enhancement in EMI SE resulted from the highly ordered alignment of the MXene nanosheets. The synergistic concurrent enhancement in the strength and EMI SE of the DC-coated MXene film can facilitate the utilization of the MXene film in reliable, practical applications.

## 1. Introduction

Two-dimensional Ti_3_C_2_T_x_ MXene nanosheets were synthesized by etching the precursor of Ti_3_AlC_2_ in a mixture of lithium fluoride and hydrochloric acid solution by Gogotsi and co-workers in 2011 [1,2]. Ti_3_C_2_T_x_ MXene nanosheets have been intensively explored in various applications such as batteries, wearable electronics, sensors, EMI materials, and thermal management because of their outstanding properties, including high electrical conductivity (10^6^ S·m^−1^), thermal conductivity (>200 W·m^−1^·K^−1^) [3,4], mechanical strength (Young’s moduli of 0.33 TPa) [5], and optical and electrochemical properties [6,7,8]. Interestingly, Ti_3_C_2_T_x_ MXene nanosheets were modified by oxygen-containing groups and/or F groups using an acidic etching approach to generate the hydrophilic characteristics of MXene, endowing aqueous solution processability by vacuum-assisted filtration [9], blade casting [10], ultra spraying [11], layer-by-layer coating [12], and drop-casting approaches [13]. Additionally, Ko et al. functionalized MXene with dual functional organic molecules composed of a catechol head and fluorinated alkyl tail which facilitated the dispersion of MXene in various organic solvents [14].

With the rapid innovation in wireless electronics, the generated EM radiation pollution has been demonstrated to deteriorate human health. Meanwhile, the bombing of micro-intelligent and portable electronic devices expects higher requirements for EMI shielding materials, e.g., lightweight and flexible [9]. Multifunctional Ti_3_C_2_T_x_ MXene nanomaterials exhibit significant advantages (large aspect ratio, high electrical conductivity, excellent thermal conductivity, facile fabrication) in these applications. Unfortunately, freestanding Ti_3_C_2_T_x_ MXene films, typically fabricated using the vacuum-filtration approach, exhibit extremely weak mechanical properties (Young’s modulus < 50 MPa) due to their brittle nature [9]. In addition, this observation could also be ascribed to the weak interactions (physical interactions or hydrogen bonding) between the flakes. This, in turn, may form a defective film structure that contains gaps and disordered alignment of flakes, causing the deterioration of the mechanical strength [15,16]. Therefore, there is a high demand for improving the interfacial interactions between MX sheets to fabricate MX films with outstanding mechanical strength. Additionally, the nature of MXene is easily oxidative in an ambient condition which hinders its industrial applications.

Strikingly, mussel byssus macromolecules are demonstrated to have a strong adhesive force on various surfaces due to the formation of non-covalent or covalent bonding interactions. Additionally, it plays a role as the “mortar” layer in the nacre structure. Recently, polydopamine, a typical polymer inspired by mussel byssus protein, has been intensively utilized as the phase for interfacial interaction in the preparation of biomimetic composites [17,18,19]. However, these strategies are complex processes with many steps which are applied to fabricate the corresponding materials. Additionally, the in situ synthesis of the polydopamine is time-consuming, which reduces the efficiency of the material manufacture. Unlike polydopamine, dicatechol-6 (DC), a mussel byssus protein macromolecule, is easily synthesized. The dicatechol-6 molecule also provides a strong adhesion with various surfaces, and it can effectively protect the surfaces from oxidation. To the best of the author’s knowledge, the modification of dicatechol-6 with 2D materials, e.g., MXene, has no report.

**Scheme 1:**
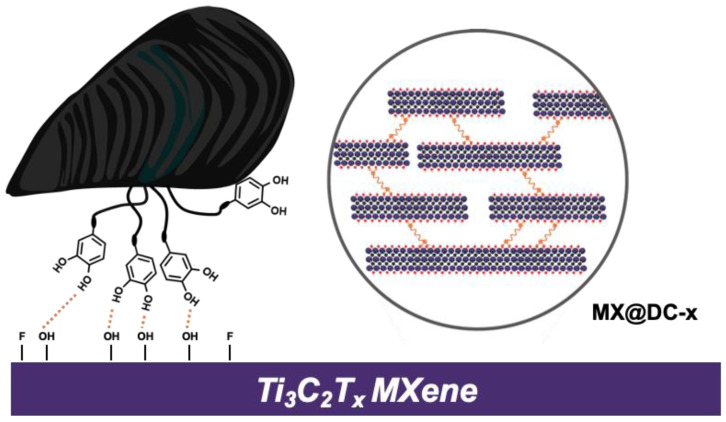
The proposed interaction between dicatechol-6 and MXene surfaces.

In this study, we designed and synthesized a mussel-like molecular structure of dicatechol-6 as an interfacial reinforcing layer for MXene films (Scheme 1). DC is synthesized by esterification between 3-(3,4-Dihydroxyphenyl)-L-alanine and 1,6-hexanediol. The MX film, which was prepared by the vacuum-filtration method, was then immersed in DC dissolved in ethanol solution. The two catechol groups of DC physically crosslinked the MX sheets and formed the brick-mortar structure of the MX@DC film. Consequently, DC molecules can generate hydrogen bonding and metal ligands with functionalities on the MXene surface, efficiently enhancing the interfacial interactions and compacted alignment of the MX sheets in the MX@DC films. The obtained MX@DC films synergistically improved the mechanical flexibility and EMI SE simultaneously.

## 2. Materials and Methods

### 2.1. Materials

3-(3,4-Dihydroxyphenyl)-L-alanine (L-DOPA) was purchased from Sejin CI Co. (Tokyo, Japan). 1,6-Hexanediol and *p*-toluenesulfonic acid monohydrate (PTSA) were obtained from Merck (Rahway, NJ, USA). Anhydrous toluene and lithium fluoride (LiF) were obtained from Alfa Aesar (Waltham, MA, USA). The Ti_3_AlC_2_ (MAX) powder was obtained from LEAP Chem Co. (Hongkong, China), and Hydrochloric acid (HCl, 37 wt.%) was purchased from Samchun (Seoul, Republic of Korea). All the reagents were used without further purification.

### 2.2. Synthesis of Dicatechol-6 (DC)

DC was synthesized as previously described [17]. L-DOPA (4.205 g, 21.3 mmol) and 1,6-hexanediol (1.20 g, 10.2 mmol) were added to a 250 mL round flask equipped with a Dean-Stark trap and a reflux condenser. The *p*-toluenesulfonic acid monohydrate (3.672 g, 21.3 mmol) and anhydrous toluene (100 mL) were added to the above solution, and the solution was stirred at 100 °C for 3 days in a nitrogen environment. The mixture was then distilled to remove residual toluene, and a pink solid product (DC, 5.5 g, yield 54.12%) was obtained without further purification.

^1^H-NMR (400 MHz, DMSO-*d_6_*) δ 8.91 (s, 4H, hydroxyl), 8.28 (s, 4H, amine), 7.48–7.46 (d, *J* = 8.1 Hz, 4H), 7.14–7.08 (d, 4H), 6.67–6.65 (d, *J* = 8.0 Hz, 2H), 6.62–6.61 (d, 2H), 6.47–6.42 (dd, *J* = 8.0 Hz, 2H), 4.23–4.13 (m, 2H), 4.12–4.00 (m, *J* = 6.2, 4.4 Hz, 4H), 2.99–2.80 (m, 4H), 2.28 (s, 6H), 1.55–1.36 (m, 4H), 1.29–1.12 (m, 4H); ^13^C-NMR (101 MHz, DMSO-*d_6_*) δ 169.15, 145.23, 145.00, 144.60, 138.04, 128.18, 125.46, 124.79, 120.15, 116.56, 115.56, 65.46, 53.46, 35.57, 27.68, 24.78, 20.77; HRMS (EI, *m*/*z*): [M^+^] calc. for C_24_H_32_N_2_O_8_, 477.2237; found 477.2239.

### 2.3. Fabrication of Ti_3_C_2_T_x_ MXene Film

Lithium fluoride (4.8 g) and 9 M aqueous hydrochloric acid solution (60 mL) were dissolved in a 100 mL Teflon flask and subjected to stirring at 50 °C for 30 min. Ti_3_AlC_2_ powder (3.0 g) was then slowly added, and the etching process was conducted at 50 °C for 30 h [18]. After that, deionized water was injected into the mixture and centrifuged at 3500 rpm for 5 min. The supernatant was decanted, and deionized water was added. This process was cycled until the supernatant reached pH 7. The supernatant was centrifuged at 3500 rpm for 15 min and diluted to obtain an MXene solution of 1 mg·mL^−1^. MXene (50 mL, 1 mg·mL^−1^) solution was vacuum-assisted filtrated using Anodic Aluminum Oxide (pore size of 20 nm) to obtain a wet film of MXene and vacuum dried at 150 °C for 1 day to achieve a thin film of MXene with a thickness of 15 μm.

### 2.4. Fabrication of Crosslinked MX@DC Films

DC was dissolved in 5 mL of ethanol by stirring at room temperature. The dried MXene film was immersed in a DC/ethanol solution for one day to perform the crosslinking reaction. The treated MXene films were rinsed with pure ethanol and vacuum dried at 150 °C. The resultant films were named MX@DC-*x*, where *x* is the concentration of the DC in the ethanol solution, which was used to immerse the MXene films, and *x* is in the range of 1–10 wt%.

### 2.5. Characterization

The chemical compositions of the dicatechol-6 and MX were determined via proton nuclear magnetic resonance spectroscopy (^1^H-NMR, ^13^C-NMR) (Bruker, Billerica, MA, USA, AVANCE 400FT-NMR), ATR-Fourier transforms infrared spectroscopy (ATR-FT-IR) (JASCO, Easton, MD, USA, FT/IR-4600), X-ray diffraction (XRD) (Bruker, Billerica, MA, USA, D2 Phaser), X-ray photoelectron spectroscopy (XPS) (PHI, Chanhassen, MN, USA, Quantera-II), and Raman spectroscopy (JASCO, Easton, MD, USA, NRS-5100). The morphology of the MXene sheet and crosslinked MX@DC-*x* films was examined using a field-emission scanning electron microscope (FE-SEM) (JEOL, Akishima, Japan, JSM-7610F). The thermal stabilities of DC and the synthesized MXene films were determined via thermogravimetric analysis (TGA) (Perkin Elmer, Middlesex, MA, USA, TGA8000). The sheet resistances were determined utilizing a 4-point probe system (AIT Co., Suwon, Republic of Korea, CMT-SR2000N). The mechanical properties were measured using a universal testing machine (Shimadzu, Kyoto, Japan, AG-250kNX). The EMI SE of the MXene films was characterized according to previous literature [9,19]. A detailed description is provided in Supplementary Materials.

## 3. Results and Discussion

### 3.1. Synthesis and Characterization of DC-Assembled MXenes

**Figure 1:**
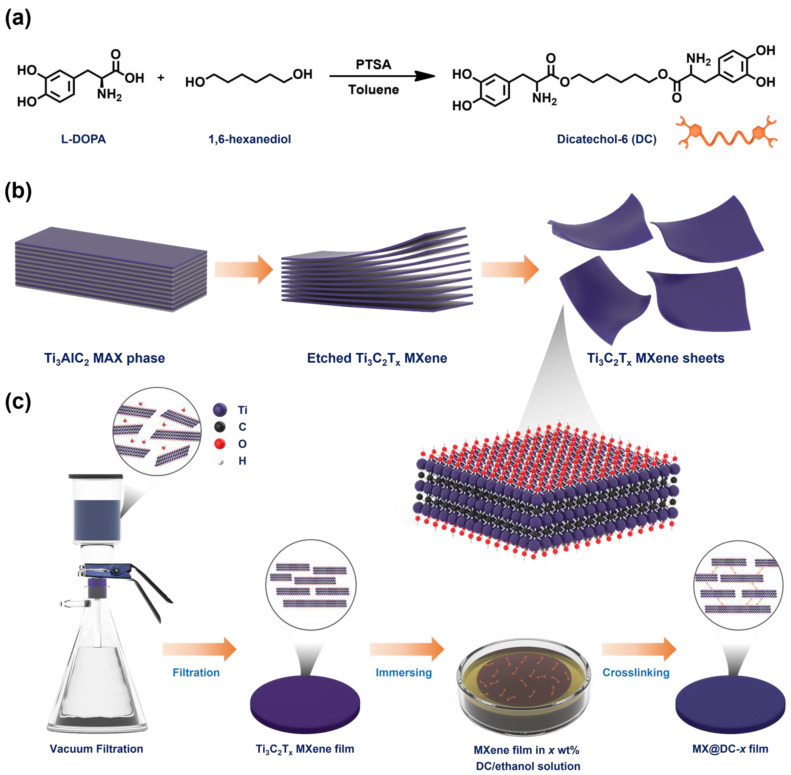
Schematic of (**a**) synthesis of DC, (**b**) fabrication of MXene, and (**c**) crosslinking MX@DC-*x* films.

The preparation of DC-Assembled MXene is presented in Figure 1. First, DC was synthesized as previously described, in which *l*-DOPA was reacted with 1,6-hexanediol while *p*-toluenesulfonic acid was used as a catalyst. The chemical composition of the synthesized DC was verified using ^1^H-NMR, ^13^C-NMR, and FT-IR. In the ^1^H-NMR spectrum (Figure S1, Supplementary Materials), there is a presence of a broad and clear -OH peak of the catechol group at 8.91 ppm, an amine peak at 8.28 ppm, the resonance pattern of the aromatic ring, and the peak of the alkyl chain of 1,6-hexanediol at 6.42–6.67 and 1.12–4.23 ppm, respectively. In the FT-IR spectra of the synthesized DC (Figure S2a,b, Supplementary Materials), the broad peak centered at 3400 cm^−1^ corresponds to -OH groups, whereas the peaks centered at 3200 and 1047 cm^−1^ are attributed to the primary amine (-NH_2_) groups and C-O groups, respectively. The peak for the methyl (-CH) groups was located at 2800–2900 cm^−1^. The C=O groups and aromatic C=C bonds can be found in the region of 1750–1730 cm^−1^ and 1600–1475 cm^−1^ [17]. The FT-IR, ^1^H-NMR, ^13^C-NMR, and Mass results confirmed that DC was successfully synthesized. TGA was used to investigate the thermal decomposition of the synthesized DC. Figure S3 (Supplementary Materials) shows that the DC can be thermally stabilized up to 280 °C. The main thermal decomposition of DC occurred in the temperature range of 270–350 °C. At 800 °C, DC char can be obtained at approximately 28 wt%. This demonstrates the high thermal stability of the synthesized DC.

**Figure 2:**
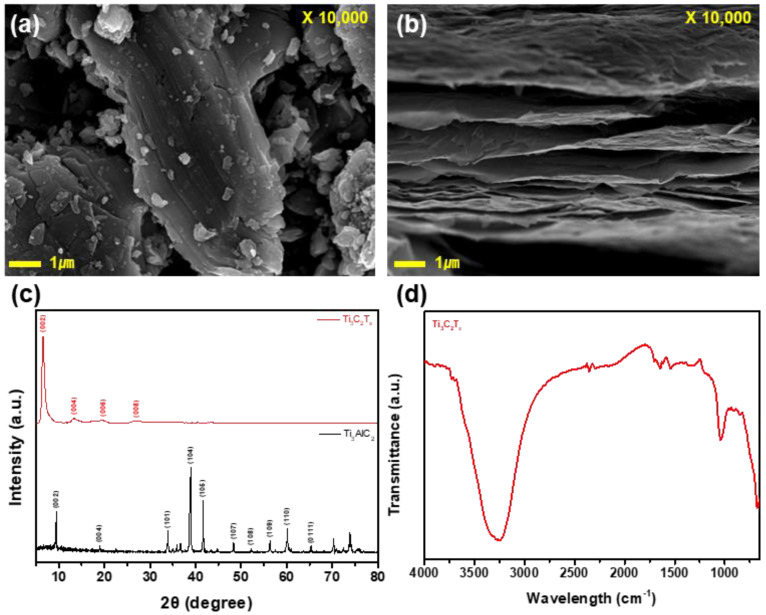
SEM images of (**a**) MAX phase, (**b**) Ti_3_C_2_T_x_ MXene, (**c**) XRD patterns, and (**d**) ATR-FT-IR spectra of the Ti_3_C_2_T_x_ MXene.

A single-layer Ti_3_C_2_T_x_ MXene was prepared by etching the aluminum layer from the Ti_3_AlC_2_, followed by vortexing for exfoliation. The morphology and chemical composition of the obtained MXene was elucidated via FE-SEM, XRD, and FT-IR. Figure 2a portrays that the bulk form of MAX has a stacked structure, and it is well exfoliated to the 2D plate-like structure of Ti_3_C_2_T_x_, which has a lateral size of microns (Figure 2b). The as-received pure Ti_3_AlC_2_ powder was phase-pure, as determined via XRD (Figure 2c). After the etching process, the characteristic peaks within the range of 33°–45° MAX were eventually eliminated. The (002) peaks shifted to 6.5° compared to that of MAX (9.5°) because of the extraction of the Al layer. The *d*-spacing of the MAX phase was approximately 0.93 nm, which was lower than that of MXene (1.35 nm). Additionally, the elimination of the (104) peak at 39° revealed the etching of Ti_3_AlC_2_ and demonstrated that the achieved MX was phase-pure [2,9,10,18]. FT-IR analysis was collected to verify the presence of functional groups. The FT-IR spectra of MX (Figure 2d) show the appearance of peaks located at 3400 cm^−1^, 1037 cm^−1,^ and 900 cm^−1^ corresponding to -OH, -C-O, and -C-F groups, respectively [1,2,18]. The EDS results (Table S1, Supplementary Materials) confirm that there is an amount of F and Cl in MXene, which were not detected in the MAX phase, while the MAX phase showed a high content of Al, which was eliminated in the MXene. This suggests that aluminum was successfully removed to form Ti_3_C_2_T_x_ MX, and there was an emergence of functional groups on the surface of Ti_3_C_2_T_x_ MX.

### 3.2. Preparation and Analysis of DC-Assembled MXene Films

**Figure 3:**
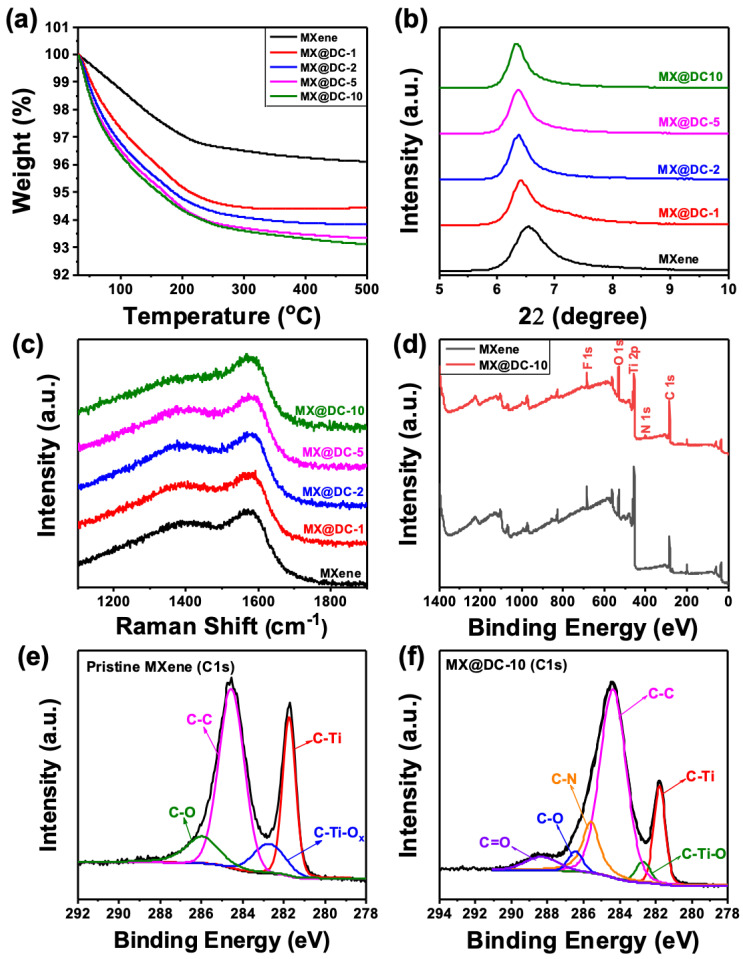
(**a**) TGA curve, (**b**) XRD patterns, and (**c**) Raman spectra of MXene and MX@DC-*x* films as a function of DC content. (**d**) Full-range XPS spectra of MXene and MX@DC-10 films. (**e**,**f**) Deconvoluted XPS C 1s of MXene and MX@DC-10 films, respectively.

The MXene solution was subjected to vacuum-assisted filtration to obtain a thin MXene film, which was then immersed in a DC/ethanol solution at different concentrations to proceed with the self-assembly and crosslinking processes between the DC and MXene sheets. Owing to the significant impact of DC loading on the strength of the crosslinked MX films, we prepared various MX@DC-*x* films. The DC content was determined using TGA (Figure 3a and Table S2 in SI). The TGA curve of the MXene film shows that it starts to decompose at low temperatures owing to the removal of moisture and non-thermally stable functional groups such as hydroxyl and fluorine [20,21]. The content of the assembled DC in the MXene films was as high as 2 wt% when the MXene film was immersed in a DC/ethanol solution with a concentration of 1 wt%. However, the higher concentration of the DC solution resulted in a slightly higher content of DC assembled on the MXene sheet surface. At a DC solution concentration of 10 wt%, the DC content in the MX@DC-10 film is roughly double that of the MX@DC-1 film. FT-IR is utilized to verify the crosslinking between MXene sheets by DC, as shown in Figure S2a,b. There are functional groups of DC in the MX@DC-10 spectra. However, the peak of the primary amine groups (3200 cm^−1^) shifts to the secondary amine groups (3300 cm^−1^), and the intensity of the hydroxyl groups decreases. The FT-IR of the MX@DC-10 shows the peaks centered at 1590 and 1100 cm^−1^ in MX@DC-10, attributing to the -N-H bonding and the -C-O-C bonding, respectively [21,22,23]. Moreover, the H-bond between the DC and the MX sheets is driven by the molecular interacted H-bond force, van der Waals force, and entropy of the hydrated layer on the surface of the MX sheets [24,25].

XRD patterns showed that the interlayer distance of the MX@DC-*x* films increased with DC loading (Figure 3b), indicating that DC was successfully assembled into the MXene sheets [26]. This is because when the DC molecules were inserted and assembled on the surface of MXene sheets, the interlayers of the MX sheets were extended, resulting in a blue shift of the (002) peak of the MX films. The *d*-spacing of the MX film was 13.51 Å and increased to 13.94 Å when the MXene film was immersed in the 10 wt% DC/ethanol solution. Raman spectroscopy was used to characterize the intensity ratio of the D-bands and G-bands of the MXene film before and after the crosslinking treatment [27]. Figure 3c and Table S2 (Supplementary Materials) show that I_D_/I_G_ decreases according to an increase in the amount of the DC loading, and that could be because the higher amount of DC was self-assembled on the MXene surface [28,29].

XPS was performed to prove the crosslinking reaction. MXene shows the appearance of Ti, C, O, and F. However, the MX@DC-10 film shows the addition of N from the DC unit (Figure 3d) [30,31]. In comparison to the high resolution of C 1s spectra of MXene in Figure 3e, the high resolution of C 1s spectra of MX@DC-10 film shows that the appearance of strong peaks of 288.5 eV and 285.8 eV, which are ascribed to the C-N and C=O stretching suggesting the presence of DC. All the mentioned evidence indicates that DC was successfully inserted into the gap between MXene sheets and MXene sheets.

### 3.3. Mechanical Properties of DC-Assembled MXene Films

**Figure 4:**
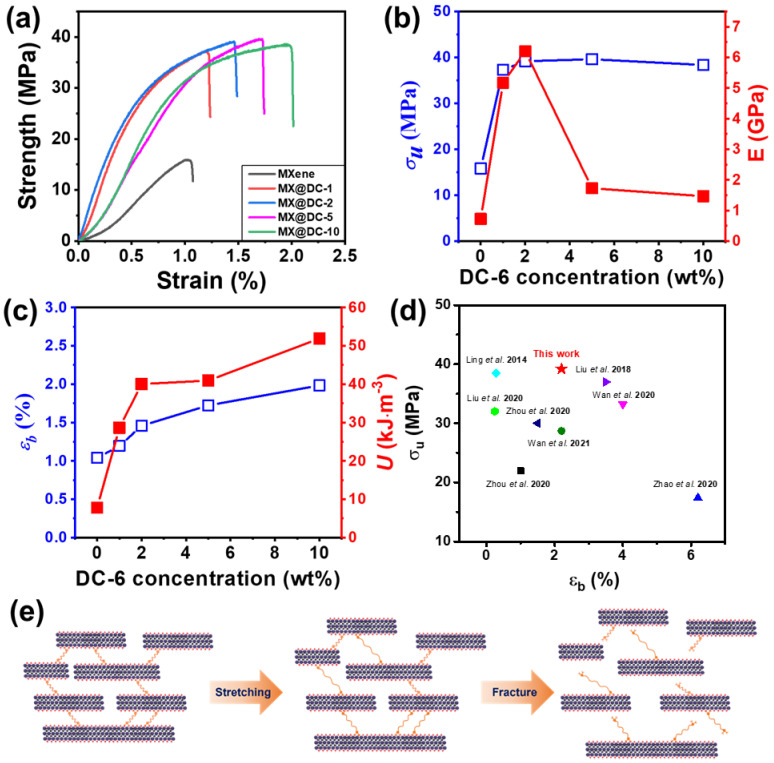
(**a**) Stress-strain curves of the MXene and MX@DC-*x* films as a function of DC content, (**b**) the ultimate strength and Young’s modulus, (**c**) elongation at break and toughness of the MXene and MX@DC-*x* films as a function of DC content, respectively. (**d**) Comparison of tensile strength of treated MXene films between our work and the previous works [28,29,41,53,54,55,56,57], (**e**) Illustration of the fracture of MX@DC-*x* film during tension test.

Excellent mechanical properties are one of the paramount requirements for the application of freestanding films [19,32]. Figure 4a–c exhibits the stress-strain curves of the pure MX films and the DC-treated MX@DC-*x* films. Overall, the pristine MXene showed weak mechanical flexibility with tensile strength (σ) of 15.83 MPa, Young’s modulus (E) of 0.73 GPa, and insignificant toughness (U) of 7.8 kJ·m^−3^. The addition of DC to MXene films facilitates an enhancement in the mechanical strength, modulus, and toughness of the MX@DC films. Notably, the MX@DC-2 films show a σ of 39.2 MPa, E of 6.2 GPa, and U of 40.02 kJ·m^−3^, which were enhanced by 2.5-fold, 8.5-fold, and 5.1-fold, respectively, compared to those of the pure MX films. Figure 4d and Table S3 (Supplementary Materials) show that our MX@DC-2 has outstanding tensile strength compared to previous studies. Figure S2b exhibits the superior flexibility of the MX@DC-5 film. The enhancement in the mechanical strengths of the MX@DC-*x* films is shown in Figure 4e. This improvement was ascribed to the strong H-bond between the hydroxyl on DC molecules and the surface of MX sheets [33,34]. The strong cohesion energy between adjacent MX sheets can be obtained owing to the strong H-bonding interactions, thus enhancing the mechanical strength [10]. Additionally, the dynamic H-bonding interactions between interlayers could diminish the energy from external stress leading to an improvement in the elongation at break. The large size of the Ti_3_C_2_T_x_ nanosheets is attributed to the high strength of the films [13,20,21]. Strikingly, a high amount of DC in the MXene films resulted in a significant increase in the elongation at break and toughness, while a reduction in Young’s modulus was observed. For example, the MX@DC-10 films exhibit breaking strain and toughness values of 1.98% and 51.91 kJ·m^−3^, respectively, which are nearly double and 6.7-fold higher than those of the pristine MXene film. Additionally, Young’s modulus of the MX@DC-10 films reduces to 1.47 GPa, which is 4.2 times lower than that of the MX@DC-2 films.

### 3.4. Electrical Conductivity and Electromagnetic Shielding Properties

**Figure 5:**
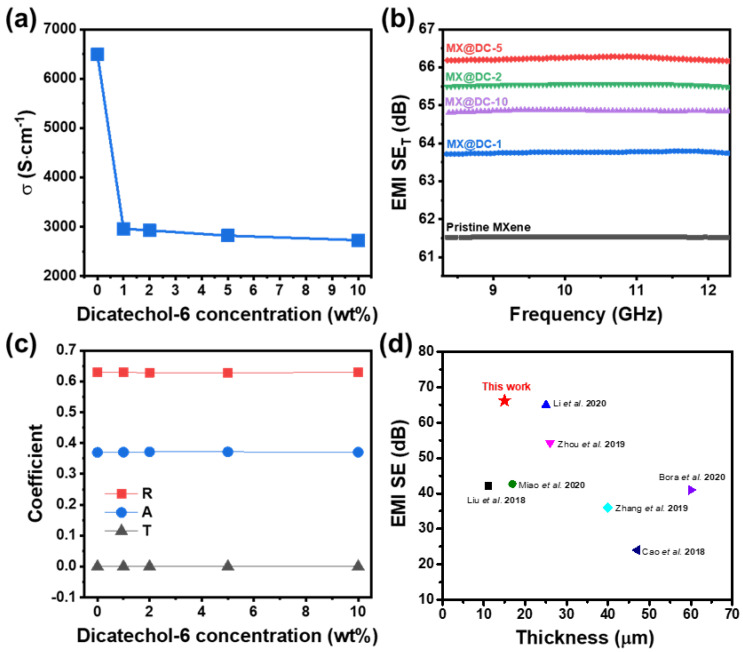
(**a**) Electrical conductivity, (**b**) EMI SE_T_, (**c**) Coefficient of reflection (R), absorption (A), and transmission (T) of the pure MXene and MX@DC-*x* films, respectively, (**d**) Comparison on EMI SE of the MXene films between our work and previous literatures [29,40,58,59,60,61,62].

Electrical conductivity undoubtedly is an important part of EMI shielding materials [35,36,37]. Figure 5a shows the in-plane electrical conductivities of the MXene and MX@DC-*x* films which were characterized using the four-probe technique. The in-plane electrical conductivity of the pure MX film was 6491 S·cm^−1^ [9]. The DC molecules on the MXene films significantly reduced the in-plane electrical conductivities of the MX@DC-*x* films. For instance, the MX@DC-1 film shows an in-plane electrical conductivity of 2960 S·cm^−1^, which is nearly 120% lower than that of the pristine MXene film. However, a higher DC loading on the MXene surface does not substantially decrease the in-plane electrical conductivity. The MX@DC-10 film, for example, exhibits an in-plane electrical conductivity of 2728 S·cm^−1^, which is approximately 1.4% lower than that of the MX@DC-2 film. It is speculated that the decline in the in-plane electrical conductivity of MX@DC-*x* can be attributed to the high amount of electrically insulating DC molecules coated on the MXene surface. However, our MX@DC-5 still has a comparable in-plane electrical conductivity compared to previous studies, which laminated MXene with a polymer matrix or nanofibers (aramid or cellulose) [38,39,40,41,42,43,44,45,46].

It is demonstrated that the MXene film shows outstanding EMI shielding performance because of its extraordinary electrical conductivity, and its surface contains abundant functionality that creates large amounts of charge carriers and forms strong dipoles. Additionally, the extremely thin (below skin depth) MXene film could also manifest excellent EMI SE owing to its lamination structure, which creates internal reflections throughout the MXene film. The high in-plane electrical conductivity is demonstrated to exhibit outstanding EMI shielding performance [47]. Figure 5b shows that the pristine MXene film showed an EMI SE of 61.5 dB, which is competitive compared to previous studies [40,41,46]. Meanwhile, MX@DC-5 showed an improvement of 7.6% in the EMI SE (66.2 dB) in comparison to the pure MXene film. Lee et al. [21] demonstrated that MXene nanosheets in MXene films prepared by the vacuum-assisted filtration method were not orderly aligned in the horizontal direction. They showed that the coating of polydopamine on the MXene surface would result in a highly ordered alignment of MXene nanosheets in the MXene film owning to the hydrogen bonding between the polydopamine molecules. Consequently, the obtained polydopamine-treated MXene films showed a higher EMI SE than the pristine MXene film [21]. We speculate that, in our study, the DC-treated MXene nanosheets could provide a highly ordered alignment, resulting in a higher EMI SE of the MX@DC-*x* films compared to that of the pure MXene film. The high content of DC loading results in a lower EMI SE due to the fact that the electrically insulating layer reduces the electrical conductivity of films. The MX@DC-10, for example, possesses an EMI SE of 65.5 dB, which is still higher than that of the pristine MXene film.

To elucidate the mechanism of EMI SE of the MX@DC-*x* films, the reflection and absorption of EMI SE, namely SE_R_ and SE_A_, were calculated, respectively. The EMI SE_R_ is ascribed to the difference in the impedances of air and material, while the EMI SE_A_ is related to the permittivity [48]. Figure S4 (Supplementary Materials) shows that the SE_A_ of the MX@DC-*x* films enhanced as increasing in the amount of DC, while the SE_R_ of the films remained constant at approximately 4.30 dB. The SE_A_ of the pristine MXene was 57.2 dB and increased to 60.5 dB for the MX@DC-2 film. Figure 5c exhibits the power coefficients of transmission (T), reflection (R), and absorption (A) of the MX@DC films as a function of the DC content. Notably, the reflection mainly contributed to shielding the power compared to that of the absorption [32,49,50]. In the MX@DC-5 film, for instance, 62.8464% of power is firstly shielded by reflection (4.3 dB of SE_R_), and then the remaining 37.1535% of the incident power is absorbed (61.9 dB of SE_A_) by the EMI shielding material. Thus, 99.9999% of the EM radiation (SE_T_ of 66.2 dB) was diminished, whereas 0.0001% was transmitted through the films. The mechanism of EMI SE of the MX@DC films can be speculated as follows: (1) due to the large mismatch of the impedance between air and the film, a large amount of EM energy of the incident EM radiation is reflected when it interacts with the surface of the films; (2) the remaining EM radiation can penetrate inside the film and absorb into the MX nanosheets resulting in a decline in the energy of EM waves [51,52]. The highly aligned order of the 2D MXene nanosheet promoted multiple reflections, resulting in effective absorption and elimination of the EM wave energy. Figure 5d and Table S4 (Supplementary Materials) show that our MX@DC exhibits a competitive EMI SE at an ultrathin thickness compared to previous studies.

## 4. Conclusions

In this paper, we report a feasible method for reinforcing the mechanical strength and EMI SE of MX films. First, a mussel-inspired molecule of DC was synthesized by esterification of 1,6-hexanediol and 3-(3,4-Dihydroxyphenyl)-L-alanine. Diluted DC in ethanol solution at various concentrations was used to immerse the laminated MX film, which was prepared by the vacuum-filtration method. The content of DC on the MXene surface increased with increasing concentration of the DC/ethanol solution from 1 wt% to 10 wt%. The DC molecules crosslink the MXene nanosheets through hydrogen bonding with the MXene surface functional groups. The obtained MX@DC films exhibited a brick-mortar structure, which improved their mechanical properties. The MX@DC-2 film, for instance, in which the MXene film is dipped in a DC solution of 2 wt%, reveals an ultimate σ of 39.2 MPa, E of 6.2 GPa, and U of 40.02 kJ·m^−3^, which is 247%, 849%, and 513% enhancement in comparison to the bare MX films, respectively. The reinforcement of the mechanical properties of the MX@DC film was ascribed to the strong interaction between the DC layers on the MXene surface. Furthermore, the higher amount of electrically insulating DC coating on the MXene surface caused a two-fold reduction in the in-plane electrical conductivity. However, the DC-coated MXene film presents a strengthening in EMI SE, with the highest EMI SE of 66.2 for MXene, which is treated in the DC solution with a concentration of 5 wt%. The enhanced EMI SE is owing to the high alignment of the MXene nanosheets and the introduction of strong dipoles and charge carriers from the DC moisture. The resulting MX@DC film, with excellent mechanical flexibility and EMI SE, can facilitate advanced industrial applications.

## Data Availability

The data that support the findings of this study can become available by the corresponding authors upon reasonable request.

## Supplementary Materials

The following supporting information can be downloaded at: https://www.mdpi.com/article/10.3390/nano13050787/s1, Figure S1: (a) ^1^H-NMR spectra and (b) ^13^C-NMR spectra of the synthesized DC in DMSO-*d_6_*. The asterisk means the residual impurities. Figure S2: (a) FT-IR spectra of DC, pristine MXene and MX@DC-10 films and (b) Digital photograph of the MX@DC-5 film which was shaped into a boat. Figure S3: TGA curve of the synthesized DC. Figure S4: EMI SE of reflection (SER) and absorption (SEA) of the MX@DC-x films as a function of DC concentration. Table S1: EDS of MAX and MXene. Table S2: I_D_/I_G_ ratio of MXene and MX@DC-x films. Table S3: A summary on tensile and strain of the pure and functionalized Ti_3_C_2_T_x_ (MXene) films. Table S4: A summary on EMI SE of the pure and functionalized Ti_3_C_2_T_x_ (MXene) films.
