# Supplementary material for: Improved Mechanical Strength of Dicatechol Crosslinked MXene Films for Electromagnetic Interference Shielding Performance
Source: Nanomaterials (Basel). 2023 Feb 21;13(5):787. doi: 10.3390/nano13050787 (PMC10005341; doi:10.3390/nano13050787)
Supplement: Supplementary file 1 [file nanomaterials-13-00787-s001.zip › nanomaterials-2218754-supplementary.pdf]

# Supplementary Materials

## Improved Mechanical Strength of Dicatechol Crosslinked MXene Films for Electromagnetic Interference Shielding Performance

Soyeon Kim[a,1], Canh Minh Vu[b,1], Suehyeun Kim[a], Insik In[a,c,*], Jihoon Lee[a,c,*]

[a]*Department of IT·Energy Convergence (BK21 FOUR), Korea National University of Transportation, Chungju 27469, Republic of Korea.*

[b]*Advanced Institue of Science and Technology, The University of Da Nang, Da Nang 550000, Viet Nam.*

[c]*Department of Polymer Science and Engineering, Korea National University of Transportation, Chungju 27469, Republic of Korea.*

[1]The authors are contributed equally to this work.

*Corresponding authors: Insik In (in1@ut.ac.kr) and Jihoon Lee (jihoonli@ut.ac.kr).

The EMI shielding performance was measured by using a vector network analyzer (E5063A ENA, Keysight Technologies, USA) with an airline kit (85051BR03) in the frequency range of 2−18 GHz based on the coaxial method. The samples were cut into a rectangular shape (with inner and outer diameters of 2 and 3 mm, respectively). The samples were sandwiched between two rectangular sample flanges. The scattering parameters ($S_{11}$ and $S_{21}$) were collected to calculate the coefficients of reflection ($R$) and absorption ($A$). Finally, the shielding effectiveness of reflection ($SE_R$), absorption ($SE_A$) and total shielding effectiveness ($SE_T$) were determined using the following equations:

$$R = |S_{11}|^2 \quad (1)$$

$$T = |S_{21}|^2 \quad (2)$$

$$A = 1 - R - T \quad (3)$$

$$SE_R = -10\ \log_{10}(1 - R) \quad (4)$$

$$SE_A = -10\ \log_{10}[T/(1 - R)] \quad (5)$$

$$SE_T = -10\ \log_{10}(T) \quad (6)$$

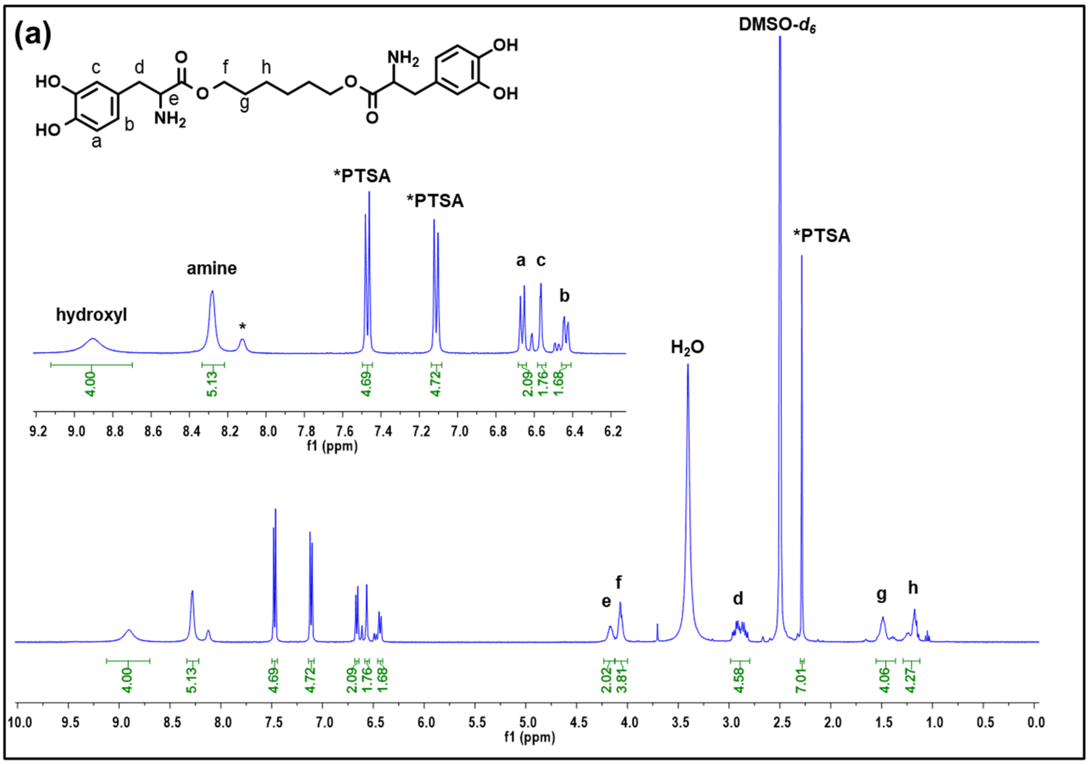


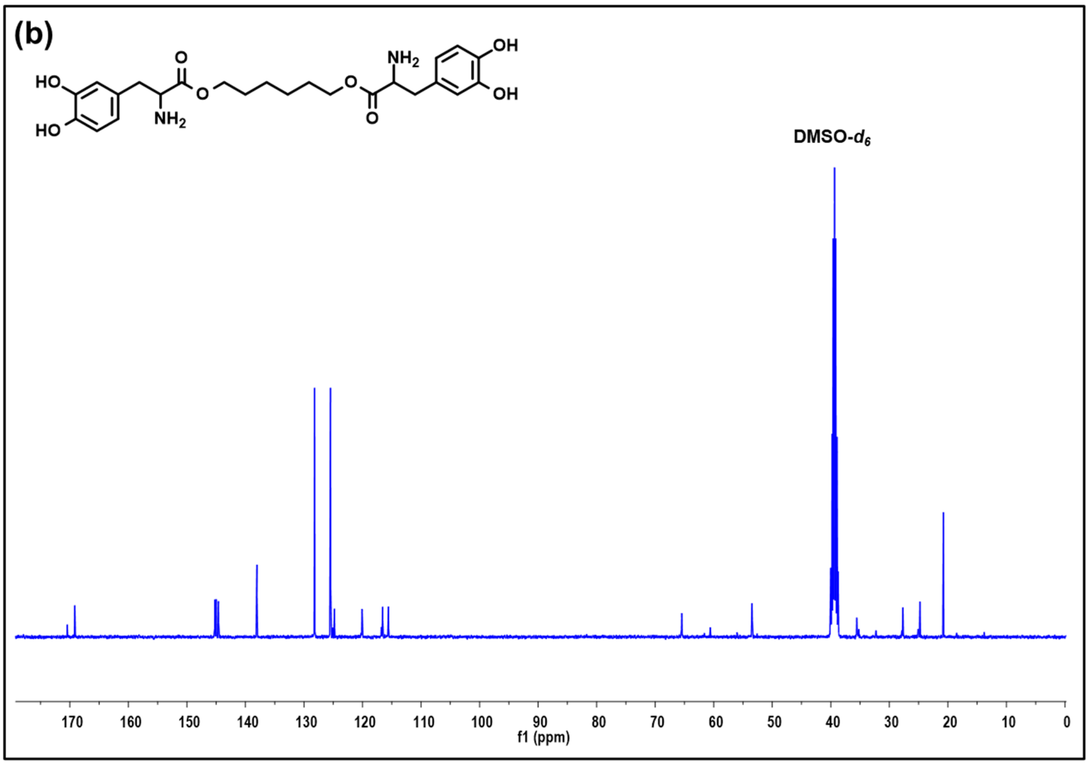


**Figure S1.** (a) $^1$H−NMR and (b) $^{13}$C−NMR spectra of the synthesized DC in DMSO−$d_6$. The asterisk means the residual chloroform and PTSA.

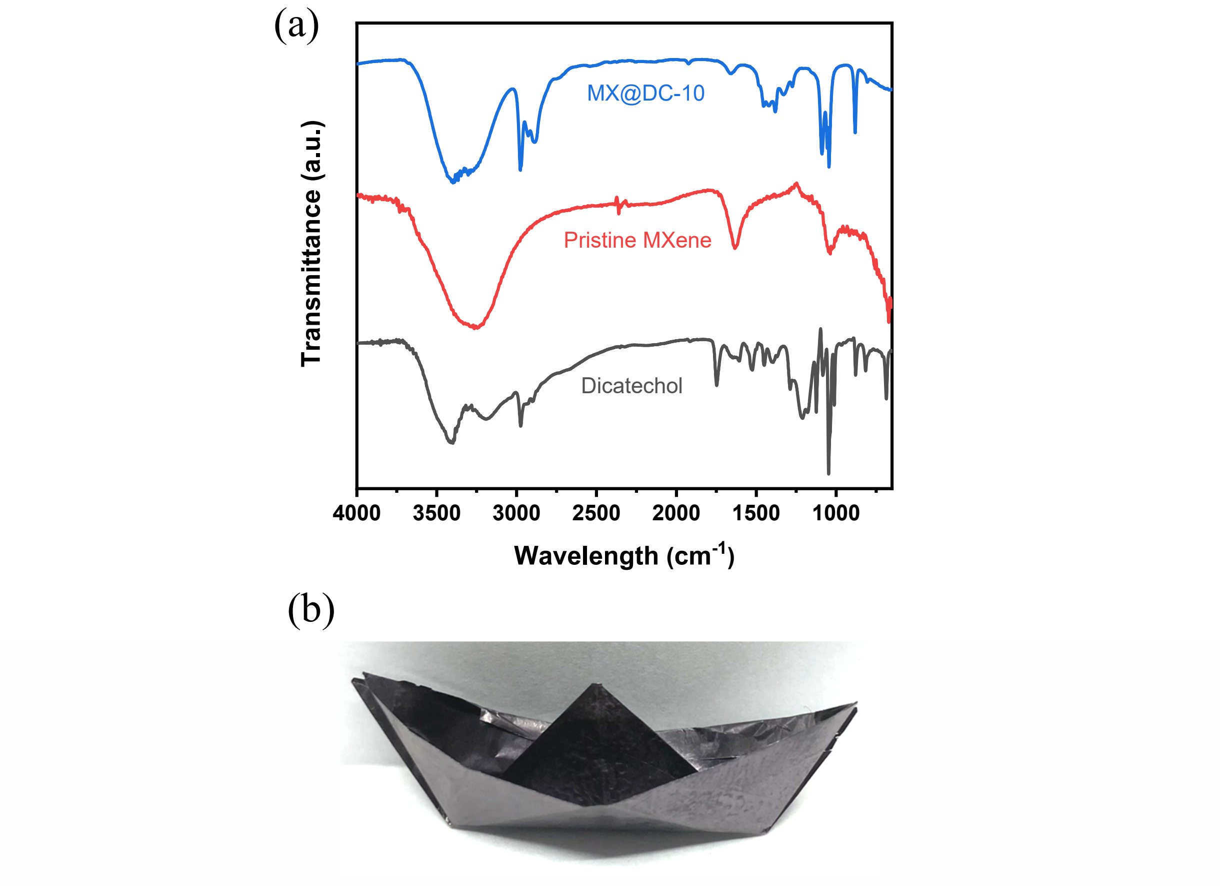


**Figure S2.** (a) FT−IR spectra of DC, pristine MXene and MX@DC−10 films, and (b) Digital photograph of the MX@DC−5 film which was shaped into a boat.

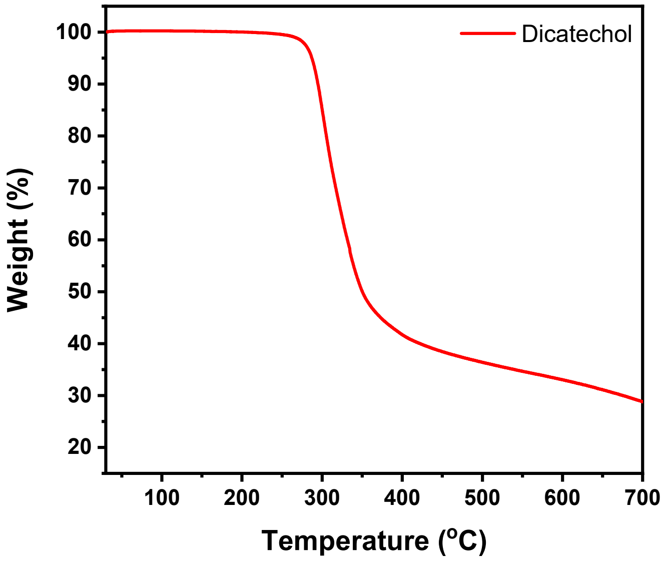


**Figure S3.** TGA curve of the synthesized DC.

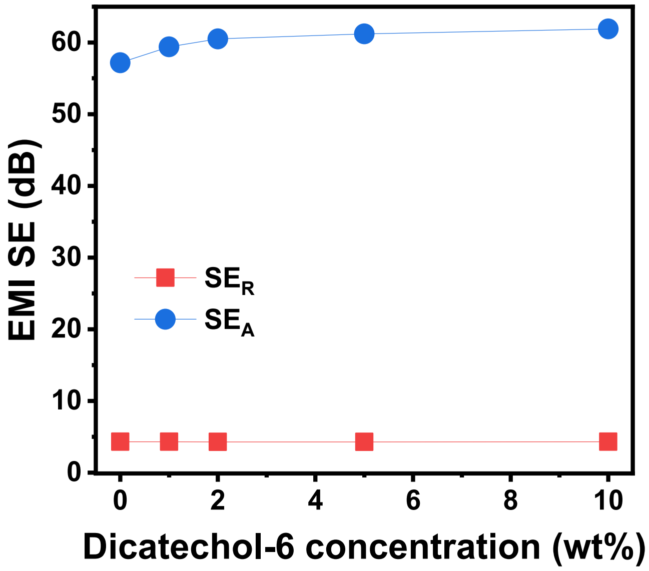


**Figure S4.** EMI SE of reflection ($SE_R$) and absorption ($SE_A$) of the MX@DC−x films as a function of DC concentration.

**Table S1.** EDS of MAX and MXene.

| Element | wt% | |
|---|---|---|
| | **MAX** | **Mxene** |
| Ti | 60.18 | 52.47 |
| Al | 14.92 | 0.19 |
| C | 15.25 | 19.46 |
| O | 9.65 | 18.9 |
| F | - | 6.18 |
| Cl | - | 2.8 |

**Table S2.** $I_D/I_G$ ratio of MXene and MX@DC−*x* films.

| Films | $I_D/I_G$ |
|---|---|
| Pristine MXene | 0.931 |
| MX@DC-1 | 0.936 |
| MX@DC-2 | 0.928 |
| MX@DC-5 | 0.923 |
| MX@DC-10 | 0.892 |

**Table S3.** A summary on tensile and strain of the pure and functionalized $Ti_3C_2T_x$ (MXene) films.

| Films | Stress (MPa) | Strain (%) | Ref. |
|---|---|---|---|
| Pure $Ti_3C_2T_x$ | 22 | 1 | Ling *et al.*[53] |
| Pure $Ti_3C_2T_x$ | 28.7 | 2.2 | Liu *et al.*[54] |
| Pure $Ti_3C_2T_x$ | 17.4 | 6.2 | Zhou *et al.*[41] |
| PANI@ $Ti_3C_2T_x$ | 33.2 | 4 | Zhou *et al.*[55] |
| PEDOT-PSS@ $Ti_3C_2T_x$ | 38.5 | 0.28 | Wan *et al.*[56] |
| PEDOT-PSS@ $Ti_3C_2T_x$ | 30 | 1.5 | Liu *et al.*[29] |
| Pure $Ti_3C_2T_x$ | 37 | 3.5 | Wan *et al.*[28] |
| Heated $Ti_3C_2T_x$ | 32 | 0.24 | Zhao *et al.*[57] |
| DC@$Ti_3C_2T_x$ | 39.2 | 2.2 | This work |

**Table S4.** A summary on EMI SE of the pure and functionalized $Ti_3C_2T_x$ (MXene) films.

| Films | EMI SE (dB) | Thickness (μm) | Ref. |
|---|---|---|---|
| $Ti_3C_2T_x$/PEDOT:PSS | 42.1 | 11.1 | Liu *et al.*[29] |
| $Ti_3C_2T_x$/AgNW | 42.7 | 16.9 | Miao *et al.*[58] |
| $Ti_3C_2T_x$/CA Aerogel | 54.3 | 26 | Zhou *et al.*[59] |
| $Ti_3C_2T_x$/MMT | 65 | 25 | Li *et al.*[60] |
| $Ti_3C_2T_x$/c-PANI | 36 | 40 | Zhang *et al.*[61] |
| PEDOT:PSS-$Ti_3C_2T_x$ | 41 | 60 | Bora *et al.*[62] |
| $Ti_3C_2T_x$/CNF | 24 | 47 | Cao *et al.*[40] |
| DC@$Ti_3C_2T_x$ | 66.2 | 15 | This work |
